# G-Protein-Coupled Receptors in Chronic Kidney Disease Induced by Hypertension and Diabetes

**DOI:** 10.3390/cells14100729

**Published:** 2025-05-16

**Authors:** Huidi Tang, Kang Li, Zhan Shi, Jichao Wu

**Affiliations:** Department of Pharmacology, Shandong University School of Medicine, Jinan 250012, China

**Keywords:** hypertension, hypertensive kidney disease, GPCR, diabetic nephropathy

## Abstract

Hypertension and diabetes are two common causes of chronic kidney disease. Hypertension can induce renal vascular injury, glomerular damage, podocyte loss, and tubular injury, leading to tubulointerstitial fibrosis. A number of factors influence the regulation of hypertension, among which G-protein-coupled receptors (GPCRs) have been studied extensively because they are desirable targets for drug development. Compared to hypertension, the regulatory effects of GPCRs on hypertensive kidney disease (HKD) are less generalized. In this review, we discussed the GPCRs involved in hypertensive kidney disease, such as angiotensin II receptors (AT1R and AT2R), Mas receptor (MasR), Mas-related G-protein-coupled receptor member D (MrgD), relaxin family receptor 1 (RXFP1), adenosine receptors (A_1_, A_2A_, A_2B_, and A_3_), purinergic P2Y receptors, and endothelin receptors (ET_A_ and ET_B_). The progression of HKD is rarely reversed but can be retarded by ameliorating the hypertensive microenvironment in the kidneys. However, simply reducing blood pressure cannot stop the progression of HKD. Diabetic nephropathy (DN) is the most common cause of end-stage renal disease (ESRD), which is a major cause of morbidity and mortality in diabetes. Many GPCRs are involved in DN. Here, we select some well-studied GPCRs that are directly associated with the pathogenesis of DN to illustrate their mechanisms. The main purpose of this review is to provide an overview of the GPCRs involved in the occurrence and progression of HKD and DN and their probable pathophysiological mechanisms, which we hope will help in developing new therapeutic strategies.

## 1. Introduction

Approximately 30% of the adult population suffers from hypertension, resulting in a large number of patients having kidney damage. Hypertension is also prevalent in people with chronic kidney disease (CKD) [1]. Therefore, hypertension presents as a common risk factor accelerating the progression of CKD, and CKD also complicates blood pressure control. The interaction between hypertension and CKD generates a vicious cycle. Different anatomical parts of the kidney with distinct cell types are mostly affected by hypertension, including vascular endothelial cells (VECs), smooth muscle cells (VSMCs), monocytes, interstitial fibroblasts, and immune cells [2]. In hypertension, renal afferent arteriolar hyalinosis accompanied by stenosis of the afferent arteriolar lumen is considered to be associated with ischemic injury of the connected glomeruli [3]. Capillary hypertension affects both podocytes and VECs in glomeruli, which results in hyperfiltration, podocyte detachment, and glomerular hypertrophy. The changes induced by intraglomerular pressure in glomeruli lead to podocyte loss, proteinuria, and, consequently, glomerulosclerosis. In addition, renal VEC injury can trigger inflammation and attract various inflammatory cells for infiltration. Hypertension-induced hypoxia, which is caused by glomerular ischemia, is the initial cause of interstitial damage [4]. Hypoxia-inducible factor α (HIFα) is induced in the tubulointerstitium, which increases the expression of collagens and other extracellular matrix (ECM) proteins, thus leading to fibrosis.

DN is the most common precipitating factor of end-stage renal disease (ESRD), and approximately one-third of the patients with diabetes worldwide show evidence of DN [5]. Among the long-term complications of diabetes, DN increases its financial burden and affects daily life. The pathogenesis of DN is complex and involves a multitude of different pathways. DN is primarily characterized by glomerular injury, such as mesangial expansion, glomerular basement membrane thickening, and glomerular sclerosis [6].

G-protein-coupled receptors (GPCRs) are one of the largest groups of proteins in the mammalian genome. The ligands for the GPCRs are largely multifarious, including proteins, ions, organic nucleotides, odorants, amines, peptides, lipids, and even photons, and can communicate their message to numerous systems and organs. The GPCRs themselves are also highly variable. All GPCRs share seven transmembrane-spanning α-helices that span the plasma membrane in a counter-clockwise manner, an extracellular N terminus that enables an extracellular ligand to exert a specific effect on the cell, an intracellular C terminus, and three interhelical loops. In addition, GPCRs are required to interact with a G-protein [7].

## 2. GPCRs in HKD

**Figure 1:**
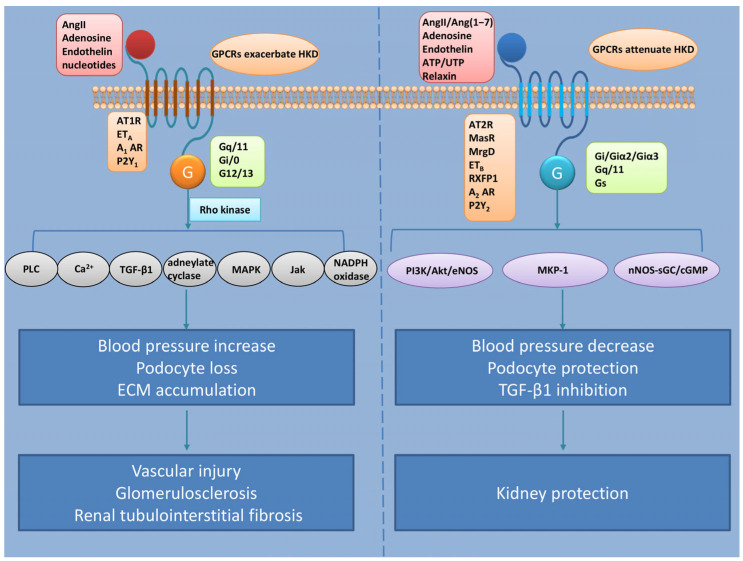
GPCRs in the pathogenesis of HKD. The left panel shows pathogenic pathways. AngII, adenosine, endothelin, and nucleotides activate receptors, including AT1R, ET_A_, A_1_AR, and P2Y_1_, which couple with G-protein subtypes (Gq/11, Gi/0, and G12/13) to drive downstream effector molecules such as Rho kinase, PLC/Ca^2+^, TGF-β1, adenylate cyclase, MAPK, Jak, and NADPH oxidase. These pathways increase blood pressure, podocyte loss, and ECM accumulation, ultimately leading to vascular injury, glomerulosclerosis, and renal tubulointerstitial fibrosis. The right panel shows protective pathways. AngII/Ang(1–7), adenosine, endothelin, relaxin, and ATP/UTP activate receptors including AT2R, MasR, MrgD, ET_B_, RXFP1, A_2_AR, and P2Y_2_. These receptors couple with Gq/11, Gi/Giα2/Giα3, and Gs proteins to activate the PI3K/Akt/eNOS, nNOS-sGC/cGMP, and MKP-1 pathways. Simultaneously, they suppress TGF-β1 signaling, exerting antihypertensive effects and podocyte-protective actions to mitigate renal damage.

Although a number of GPCRs have been demonstrated to be involved in the genesis and progression of hypertension, the mechanism for the progression of hypertensive kidney disease is still obscure. Here, we discuss angiotensin II receptors (AT1R and AT2R), Mas receptor (MasR), Mas-related G-protein-coupled receptor member D (MrgD), relaxin family receptor 1 (RXFP1), adenosine receptors (A_1_, A_2A_, A_2B_, and A_3_), purinergic P2Y receptors, and endothelin receptors (ET_A_ and ET_B_) because they play important roles in the regulation of hypertension and kidney function (Figure 1). Subsequently, when discussing important mechanisms of hypertensive kidney disease, we will focus on the GPCRs associated with different renal tissues and cell types that are affected by hypertension.

### 2.1. GPCRs Involved in Renal Vascular Injury and Glomerular Damage Under Hypertensive Conditions

Vascular injury, characterized by endothelial dysfunction, structural remodeling, fibrosis, and inflammation, is a common phenomenon in hypertension because of high pressure and blood flow shear force [8,9]. VECs are the first to be affected in hypertensive vessels, and VSMCs contribute to vascular structural remodeling induced by hypertension. With the progression of hypertension, arteriolosclerosis or hyalinosis, characterized by the intimal thickening of small arterioles or the thinning of the media, respectively, occurs. Renal function and blood pressure control are almost inseparable, and hypertension is a major risk factor for renal disease [10]. Glomerular damage caused by hypertension is mainly ascribed to afferent arteriole stenosis, which results in the partial ischemia of the glomerular tuft.

The renin–angiotensin system (RAS) is an important mediator in regulating blood pressure, vascular tone, and electrolyte balance. Two GPCRs, angiotensin type 1 receptor (AT1R) and angiotensin type 2 receptor (AT2R), are the main regulators in this system. A growing amount of evidence shows that AT1R and AT2R play a critical role in mediating hypertension and related diseases [11,12]. The activation of AT1R via binding with angiotensin II (AngII) stimulates diverse intracellular signaling pathways such as phospholipase C (PLC), Ca^2+^ channels, phospholipase D (PLD), phospholipase A2 (PLA2), adenylate cyclase, MAP kinases (MAPK), the JAK-STAT pathway, and NADPH oxidase through the coupling of G proteins (Gq/11, Gi/0, and G12/13) to the C-terminal of the receptor, which participates in cellular proliferation, fibrosis, apoptosis, and inflammation to facilitate the pathological conditions of hypertension [13]. AT1R mediates most of the pathophysiological effects of Ang II in the vascular system, such as vasoconstriction/hypertension, inflammation, the proliferation of VSMCs, and vascular fibrosis [14]. AT1R is expressed in almost all kidney cell types and exerts indirect effects via increasing filtration pressure, changing glomerular hemodynamics, increasing NaCl filtration, and consecutively adapting the tubular salt and water handling. Studies on renal cross-transplantation have shown that AT1R in the kidneys is both necessary and sufficient for the development of angiotensin II-dependent hypertension [15]. Furthermore, AngII/AT1R also increases the tubular expression of angiotensinogen, ACE, renin, and AT1R, which aggravates hypertension and kidney injury. AT2R, which is activated by binding with AngII, elicits G protein (Gi), resulting in MAPK inhibition through the activation of phosphotyrosine phosphatases [16]. Furthermore, it is believed to have counter-regulatory effects compared to AT1R, which is due to NO/cGMP activating a cGMP-dependent protein kinase, leading to RhoA activity inhibition and, consequently, suppressing AT1R-associated pathophysiological processes [17,18]. Although AT2R has been detected in low abundance in the adult kidney, it plays a key role in blood pressure control by mediating natriuresis. Despite the effects of AT1R on the holistic regulation of hypertension, AngII-activated AT1R can directly cause VEC dysfunction by promoting intracellular reactive oxygen species (ROS) generation and impairing endothelial nitric oxide synthase (eNOS)/nitric oxide (NO) function [15,19]. It can also attenuate the bioactivities of human endothelial progenitor cells via the downregulation of the β2-adrenergic receptor, which retards endothelial repair. Once endothelial integrity is disturbed, vascular remodeling is facilitated due to thrombosis, the creation of an inflammatory environment, and the growth promotion and migration of VSMCs, resulting in the degradation and reorganization of the extracellular matrix. In hypertension, AngII/AT1R-induced hyperplasia and hypertrophy also contribute to vascular remodeling by promoting a phenotypic switch from a contractile to a proliferative and synthetic phenotype of VSMCs [20]. RGS2 is one of the earliest reported RGS proteins associated with the negative regulation of AT1R signaling in VSMCs [13]. Experiments have found that, compared with wild-type mice, AngII infusion increased the blood pressure of RGS2-knockout mice to a greater extent. This is due to the increased reactive myogenic tone and vascular reactivity of AT1R activated by AngII in arterial VSMCs [21]. This indicates that RGS2 has the effect of attenuating the vasoconstrictive signaling of AT1R in VSMCs. On the contrary, the activation of AT2R promotes NO production dependent or independent of bradykinin, which may protect VECs. The NO synthase inhibitor abolishes the AT2R-mediated vasodilatory response. AT2R also negatively regulates VSMC remodeling by inhibiting the activity of RhoA/Rho kinase in angiotensin II-induced vasodilation in stroke-prone spontaneously hypertensive rats [22]. Some reports demonstrated that AT2R activation inhibits pn in vascular smooth muscle cells by stimulating MAPK phosphatase (MKP-1) and protein phosphatase 2 (PP2A) to exert its anti-remodeling actions [23]. Moreover, AT2R and its interacting protein, ATIP1, interact with each other to ameliorate vascular intimal proliferation.

MasR is another GPCR that participates in blood pressure regulation by binding with Ang(1–7). ACE2 hydrolyzes Ang II to produce Ang(1–7), which exerts a protective effect through MasR [24]. The activation of MasR increases NO production through the phosphoinositide 3-kinase (PI3K)/Akt/eNOS pathway and activates SHP-2 in endothelial cells, which counteracts AngII/AT1R-induced hypertension and VEC dysfunction [25]. However, some reports imply that the functions of MasR depend on AT2R. Ang(1–7) administration ameliorates blood pressure and proteinuria in L-NAME-treated spontaneously hypertensive rats (SHRs), which is associated with lower histological grades of hypertensive vascular injury in the kidneys [26].

MrgD, a member of the Mas-related GPCR family and also known as TGR7 or hGPCR45, was first reported in a subset of pain-sensitive small-diameter neurons, which were associated with neuropathic pain. In blood vessels, the expression of MrgD was detected in VSMCs, eNOS-positive endothelial cells, and atherosclerotic plaques. It has been demonstrated to be a negative receptor for hypertension induced by AngII/AT1R when it binds to alamandine or Ang(1–7) [27]. Studies on the modulating effects of MrgD on hypertensive kidney disease are very rare. However, the activation of MrgD by alamandine increased cAMP concentration in primary endothelial and mesangial cells. Moreover, it reversed hyperhomocysteinemia-induced vascular dysfunction in the isolated abdominal aorta of New Zealand White Rabbits through the Gi protein signaling pathway [28]. The vasodilation effects of MrgD activated by alamandine are also mediated by NO release, and oral doses of alamandine lead to a long-lasting antihypertensive effect in SHRs. Alamandine administered via a microinjection into the caudal ventrolateral medulla exerted an MrgD-dependent hypotensive effect in hypertensive rats. Ang(1–7) has pleiotropic effects in vivo, including NO-mediated vasodilation and regulation of the baroreflex, cardioprotection, and beneficial metabolic effects. In most cases, the effects of Ang(1–7) negatively regulate the effects of Ang II. Most of the effects of Ang(1–7) are absent in Mas-KO mice, and in many cases, the genetic deletion of Mas produces changes that are opposite to those induced by the administration of Ang(1–7) [24]. Although there is little evidence of MrgD participating in hypertensive kidney injury, it is a promising novel drug target for protecting the kidney through antihypertensive actions.

Relaxin family receptors 1–4 (RXFP1–4) are four GPCRs that are activated by binding with relaxin to produce their physiological effects. Among these four receptors, RXFP1 is the most explored and is involved in the regulation of blood pressure [29]. RXFP1 is the cognate receptor for human relaxin-2 in humans, widely expressed in the heart, kidneys, liver, lungs, blood vessels, and the various areas of the brain, and involved in numerous signaling pathways by increasing cAMP and NO levels in tissues [29]. Although the expression of RXFP on the VECs and/or VSMCs of the afferent and efferent arterioles has not been established, RXFP1 has previously been localized in renal interlobar arteries. Studies also report that RXFP1 is primarily expressed on VSMCs and, to a lesser extent, on macrophages and the endothelial cells of arteries and veins [30]. In a model of arteriovenous fistulas, RXFP1 deficiency aggravates vascular inflammation and impairs outward remodeling. In addition, RXFP1 protects microvasculature and attenuates inflammation following cardiac ischemia–reperfusion. In an exploratory study of human cirrhosis, serelaxin, a recombinant peptide of human relaxin-2, specifically mediated renal vasodilation by binding to RXFP1. Serelaxin was found to increase kidney perfusion and oxygenation, reduce renal vascular resistance, reverse endothelial dysfunction, and increase the activation of the AKT/eNOS/NO signaling pathway in the kidneys [31]. Xiaoming Lian and colleagues demonstrated that the relaxin-2-induced activation of RXFP1 resulted in the endothelium- and NO-dependent relaxation of mouse mesenteric arteries via coupling with the Gi_2_-PI3Kγ/β-eNOS/NO pathway, and these effects might ameliorate endothelial dysfunction in pathological states such as hypertension [32]. Short-term relaxin treatment promotes endothelium-related vasodilation by upregulating eNOS, COX2, and prostacyclin receptor expression and reverses hypertension-induced vascular dysfunction through interactions with endothelium-derived contracting factors by restoring endothelium-derived PGI2 vasodilator pathways. Relaxin had a potent dilatory effect in rodent renal and human subcutaneous arteries, and the underlying mechanisms were supposed to be associated with the activation of the PI3K/Akt and eNOS signaling pathways [33]. The short-term administration of relaxin can rapidly activate the cAMP, cGMP, and pERK1/2 signaling pathways in endothelial cells derived from human arteries and veins, VSMCs, and fibroblasts. This process is dependent on G proteins such as Gαs, Gαi/o, and GαOB and involves the downstream PI3K pathway. These signaling pathways may serve as the mechanistic basis for the rapid vasodilation induced by relaxin in human subcutaneous arteries as well as in the renal and mesenteric arteries of rodents [34]. Although there is less evidence on the protective effects of RXFP1 on hypertensive renal vascular injury, the effects of RXFP1 on blood pressure regulation, microvascular protection, and inflammation prevention convince us that relaxin/RXFP1 activation plays an important role in improving renal vascular function under a hypertensive environment.

Adenosine receptors (ARs), also called purinergic P1 receptors, include four distinct GPCR subtypes, i.e., A_1_, A_2A_, A_2B_, and A_3_, and mediate various aspects of organ function via the adenylate cyclase system. The A_1_ AR and A_3_ AR receptors inhibit the production of cAMP through coupling to Gi, while the A_2A_ AR and A_2B_ AR subtypes are coupled to Gs or Go to stimulate adenylate cyclase [35]. In the kidneys, A_1_ is located on the preglomerular vessels, and the tubules play a role in the regulation of GFR. It has been reported that A_1_ AR and AngII have a synergistic interaction to induce the constriction of the renal microvasculature by increasing intracellular Ca^2+^ sensitivity via the modulation of PLC, myosin light chain (MLC) phosphorylation, and p38 MAPK [36]. A_1_ AR participates in regulating GFR and represents synergistic effects with AngII, which possibly contribute to renal vascular injury and remodeling induced by hypertension. The activation of A_2_ AR has counter-regulatory effects compared to A_1_ AR. When adenosine is administered for intrarenal infusion in rats, vasoconstriction occurs in all cortical zones where A_1_ AR dominates, while vasodilation mediated by A_2_ AR occurs in deep cortical glomeruli. Accordingly, an A_2_ AR agonist increases the medullary blood flow by perfusing the renal interstitial substance, whereas a selective A_2_ AR antagonist decreases medullary blood flow via intramedullary infusion [37]. The activation of A_2B_ AR inhibits VSMC proliferation while promoting VSMC apoptosis through cAMP signaling. In addition, the activation of A_2A_ AR attenuates early inflammatory processes that are related to neointimal growth following vascular injury [38]. Furthermore, adenosine receptors play a critical role in regulating arteriolar resistance. A_1_ ARs are highly expressed in the afferent arteriole, where their activation inhibits adenylate cyclase activity, reduces intracellular cAMP levels, and induces vasoconstriction, thereby decreasing GFR. In contrast, A_2_ AR stimulates adenylate cyclase, increases cAMP production, and promotes vasodilation [39]. An imbalance between these two signaling pathways may further exacerbate the decline in GFR observed in hypertensive nephropathy. Though a few studies have explored the effects of A_2_ AR on hypertensive kidney disease, A_2_ AR can become a new topic because of its wide range of functions in the regulation of vascular pathophysiological characteristics.

Extracellular nucleotides activate two families of P2 receptors: P2Y and P2X. The P2Y receptors belong to GPCRs, including eight mammalian subtypes—P2Y_1_, P2Y_2_, P2Y_4_, P2Y_6_, P2Y_11_, P2Y_12_, P2Y_13_, and P2Y_14_. The eight subtypes are divided into two distinct subgroups characterized by a high level of sequence divergence, P2Y_1,2,4,6,11_ and P2Y_12,13,14_. These two subgroups couple to different G proteins. P2Y_1,2,4,6,11_ principally couples to Gq/G_11_ to increase cytosolic Ca^2+^ concentration ([Ca^2+^]i) and activate the PLC β isotype/inositol 1,4,5-trisphosphate (IP3) pathway, whereas P2Y_12,13,14_ couples with the members of the Gi/o family of G proteins to inhibit adenylyl cyclase and reduce the cAMP levels [40]. The activation of vascular and glomerular purinergic P2 receptors may contribute to the mesangial cell transformation, vascular hypertrophy, and renal inflammation in response to AngII-induced hypertension. An early study identified that the infusion of ATP or 2-methylthio ATP (P2Y agonist) to the lumen of rabbit renal arteries pre-constricted with norepinephrine yielded consistent vasorelaxation that was attenuated by the non-selective P2Y receptor, and the vasodilatory effect of ATP also appeared in human renal arteries (pre-constricted with prostaglandin F_2α_) [41]. In the following study, Rump and coworkers suggested that P2Y receptor-induced renal dilation can be mediated by NO because the vasorelaxation due to 2-methylthio ATP was largely abolished by the NO synthase inhibitor N^G^-nitro-L-arginine [42]. However, the renal afferent arteriole diameter was reduced by a high concentration of 2-methylthio ATP (100 μM). These effects of 2-methylthio ATP-activated P2Y receptors seemed to increase blood flow and enhance the perfusion pressure in nephrons, which increased GFR. In salt homeostasis, blood pressure regulation, and aldosterone escape studies, the ATP/UTP/P2Y_2_ receptor system was demonstrated to play an important role in suppressing epithelial sodium channel (ENaC) activity when sodium transport was enhanced by the high levels of mineralocorticoids in combination with the high NaCl intake, which reduced blood pressure and attenuated aldosterone escape [43]. In addition, P2Y_2_ receptors participate in the acute NO-independent blood pressure-lowering effect of Ip4U (P1-(inosine 5′-)P4-(uridine 5′-)tetraphosphate), a P2Y_2_/P2Y_4_ receptor agonist, implicating P2Y_2_ receptors in the vascular response to the endothelium-derived hyperpolarizing factor. In addition to vasodilation, P2Y_2_ receptors were implicated in the inhibition of renal sodium reabsorption.

Endothelin (ET) receptors include two subtypes, ET_A_ and ET_B_ receptors, which are mutually antagonistic in regulating vasoconstriction, cell proliferation, and inflammation. The ET_A_ receptor mainly promotes vasoconstriction, cell proliferation, and inflammation by binding with ET-1, and the ET_B_ receptor can be considered its endogenous antagonist because it inhibits the aforementioned ET-1-mediated effects [44]. In humans, GPR37 (endothelin receptor type B-like or Parkin-associated endothelin receptor-like receptor) and GPR37L1 are identified to be exactly similar in structure to ET_A_ and ET_B_. Wendel and colleagues identified that the ET_A_ receptor was expressed on both VSMCs and VECs, while the ET_B_ receptor expression was restricted to VECs in the renal vasculature [45]. An early study suggested that ET-1/ET_A_ contributes to renal hemodynamics and regulates glomerular pressure in a physiological dose [46]. In addition, ET-1/ET_A_ can reduce GFR because it is more sensitive in afferent than in efferent arterioles, and the ET_B_ receptor activation can induce NO-mediated vasodilation in efferent arterioles. Specifically blocking the ET_A_ receptor reduces blood pressure and enhances renal blood flow in hypertensive patients with chronic renal failure, but it does not affect GFR. The acute blockage of the ET_A_ receptor also attenuates proteinuria and arterial stiffness in a blood pressure-independent manner. The ET_B_ receptor, but not ET_A_, expressed in the rat proximal tubule, facilitates natriuresis by inhibiting Na^+^/K^+^-ATPase via binding with ET-1 and then enhancing Ca^2+^ recruitment [47]. The ET_B_ receptor is also the only receptor in the medullary thick ascending limb, which inhibits sodium reabsorption through the activation of PI3K, AKT, and eNOS and consecutively elevates NO production, which inhibits the Na^+^/K^+^/Cl^−^ cotransporter [48]. The ET_B_ receptor exhibits the highest density in the collecting duct compared to other cell types in the rat kidneys but expresses a low amount of ET_A_ receptors. The activation of the ET_B_ receptor in the collecting duct suppresses Na^+^/K^+^-ATPase by activating cyclooxygenase and inhibiting ENaC by promoting the guanine exchange factor βpix to bind the 14-3-3 protein and activating MAPK1/2, with a phosphorylation status of ENaC [49]. These effects of the ET_B_ receptor on salt reabsorption in the renal tubules probably contribute to reduced blood pressure in hypertension. A recent study indicated that the dual blockage of the ET_A_ and ET_B_ receptors mitigated hypertension in a rat model of CKD [50].

Sodium transport plays a key role in blood pressure control, and many GPCRs, including those mentioned above, are involved in this process, such as vasopressin receptors, catecholamine receptors, bradykinin B2 receptors, prostanoid receptors, dopamine receptors, proteinase-activated receptors 2, α-ketoglutarate receptor OXGR1, and sphingosine-1-phosphate receptors. These receptors have been well documented in a review [51].

### 2.2. GPCRs Involved in Podocyte Loss and Glomerulosclerosis Induced by Hypertension

Podocytes are the most important cells for the maintenance of the integrity of the glomerular slit diaphragm. As podocytes are terminally differentiated cells, their loss is irreversible. Glomerular hypertension is the major factor inducing podocyte injury, death, and then detachment from the glomerular basement membrane in hypertensive kidneys [52].

In accordance with the prominent glomerular histopathology in hypertensive kidney injury, the elevation of AngII results in the severe injury of podocytes with foot process effacement, ultimately facilitating podocyte loss. The blockade of Ang II has beneficial effects on the amelioration of HKD, which correlates with the attenuated podocyte loss. AngII incurs podocyte injury via the following pathways. By binding to AT1R, an intracellular cascade of kinases is activated, including the calcium–calmodulin binding, the activation of PLC, pERK, PKC, ribosomal S6 kinase (RSK), and protein kinase A (PKA), and the upregulation of cAMP. Zhao Y and colleagues provide evidence that AngII induces podocyte injury by downregulating microRNA-30 family members, providing a new insight into the mechanism of podocyte injury in hypertension [53]. In contrast, AT2R shows a protective effect on podocytes, which antagonizes AT1R. In AT2R-knockout mice, ectopic hedgehog interacting protein (Hhip) gene expression is amplified, resulting in either podocyte loss via triggering caspase-3- and p53-related apoptotic processes or by transforming podocytes to podocyte-derived fibrotic cells through the activation of TGFβ1-Smad2/3 cascades and αSMA expression.

The activation of MasR by Ang(1–7) has similar effects as AT2R on podocyte protection. It has been reported that Ang(1–7) attenuates podocyte injury induced by preeclamptic serum treatment through the downregulation of MAPK phosphorylation [54]. In addition, the podocyte injury and loss may be ascribed to decreased Ang(1–7) and downregulated intrarenal RAS in preeclampsia. Though studies of MasR involvement in podocyte injury in HKD have not been widely reported, some evidence implies its beneficial effects in protecting podocytes [55,56].

P2Y_1_ is predominantly expressed in podocytes, as demonstrated by comprehensive pharmacological profiling and immunolocalization. In the P2Y_1_-null mice, the phenotype shows reduced capillary rarefaction, preserved renal function, and fibrosis. The protective effects of P2Y_1_ inhibition on podocytes may produce a new therapeutic strategy for podocyte loss induced by hypertension [57]. Using two-photon microscopy to injure one single podocyte triggered the spreading of podocyte [Ca^2+^]i waves that depended on the presence of P2Y_2_ receptors, and in a rat anti-Thy1 model of glomerulonephritis, a transient increase in glomerular P2Y_2_ and P2Y_6_ expression was detected, together with P2 receptor-dependent (using the non-selective P2 inhibitor PPADS) mesangial cell proliferation [58].

Podocytes express both ET_A_ and ET_B_ receptors. ET receptors play an important role in podocyte injury and loss due to their moderating effects on glomerular perfusion pressure and inflammation. It has been reported that the activation of ET_A_ receptors by ET-1 infusion aggravates inflammation, resulting in podocyte effacement [59]. ET-1 is also found to contribute to synaptopodin loss in cultured mouse podocytes via recruiting β-arrestin-1. Puromycin aminonucleoside-induced podocyte injury and cytoskeleton rearrangement are abrogated by the ET_A_ receptor blockade. In homozygous hypertensive Ren-2 rats, atrasentan, a selective ET_A_ receptor blocker, reduces podocyte injury [60]. The selective blocking of ET_A_ receptors exerts profound anti-proteinuria effects even in malignant hypertension and attenuates podocyte injury prior to proteinuria. The inhibition of ET receptors by using non-selective ET receptor antagonists also reverses established glomerulosclerosis caused by chronic hypertension. Moreover, Muhammad and colleagues found that uric acid-induced glomerulosclerosis and renal fibrosis may be attributed to the upregulation of ET-1 [61]. Inhibiting ET_A_ receptors on glomerular endothelial cells is beneficial for preventing glomerular endothelial surface layer degradation due to mitochondrial ROS scavenging in adriamycin-induced glomerulosclerosis.

Although there are few studies focusing on the correlation between vasopressin receptors and HKD, vasopressin receptors (V1 and V2) are proven to be motivators of podocyte loss and glomerulosclerosis [62]. It has been reported that the antagonist of vasopressin receptors, tolvaptan, inhibits podocyte injury and glomerulosclerosis by suppressing NF-κB phosphorylation and Rho kinase, ERK, and ROS production in Dahl rats with end-stage heart failure [63]. Another antagonist, YM218, is proven to ameliorate glomerulosclerosis after 5/6 nephrectomy. In SHRs, an early study demonstrated that the nonpeptide vasopressin receptor antagonist of V1, OPC-21268, inhibited nephrosclerosis [64].

### 2.3. GPCRs Involved in Renal Tubulointerstitial Fibrosis Induced by Hypertension

Patients with hypertension may develop a large spectrum of lesions in their epithelial tubular cells [2]. Renal tubulointerstitial fibrosis is aggravated by numerous molecules, cells, and factors, such as Ang II, TGFβ, cellular senescence, inflammation, interstitial hypoxia, fibroblasts, and the epithelial–mesenchymal transition (EMT) [65,66]. The mechanisms of renal tubulointerstitial fibrosis are still poorly understood, and existing therapies are only slightly successful at reversing this process.

AngII can modulate renal tubulointerstitial fibrosis via its direct effects on the ECM recruitment and by upregulating the expression of other factors [67,68]. AngII is one of the most important factors in inducing the EMT. The EMT is a process resulting from epithelial cells losing their cell–cell adhesion to become mesenchymal cells, and it plays a crucial role in fibrosis, particularly in the kidneys [69]. Accumulating evidence has now indicated that AT2R is a kidney-protective target, shielding injured kidneys from the progression of tubulointerstitial fibrosis [70]. Although the relationship between RAS and HKD has been widely explored, the progression of tubulointerstitial fibrosis continues.

RXFP emerges as an effective antifibrotic in many different models of renal fibrosis. A study aimed at illuminating the signal transduction mechanisms by which its TGF-β1-inhibitory effects are mediated in myofibroblasts has shown that relaxin signals through RXFP1 to phosphorylated/activated ERK1/2 (pERK1/2) and the nNOS-sGC/cGMP-dependent pathway [71]. In renal myofibroblasts, relaxin exerts its anti-fibrotic actions via an NOsGC-cGMP-dependent mechanism by binding with RXFP1, which would contribute to suppressing hypertensive renal fibrosis. In 2005, a study demonstrated that relaxin reverses renal fibrosis in spontaneously hypertensive rats by normalizing collagen content of the kidneys [72]. Moreover, another report indicated that relaxin could attenuate salt-sensitive hypertension and renal fibrosis by binding to renal local RXFP1 through NOS upregulation and TGF-β1 downregulation [73]. When activated by B7–33, a relaxin analog, RXFP1 exhibits powerful anti-fibrotic activity by promoting matrix metalloproteinase 2 (MMP2) expression through ERK1/2 phosphorylation while showing low efficacy for cAMP production. A recent study reports that AT1R-AT2R-RXFP1 have functional crosstalk in myofibroblasts [74]. However, the heteromeric formation between RXFP1 and AT2R as well as AT1R and AT2R has been previously proposed [75], and researchers have provided evidence that RXFP1, AT1R, and AT2R are expressed on myofibroblasts and heteromers can form between RXFP1 and AT1R. This indicates that blocking AT1R exerts anti-fibrotic effects by activating the AT2R ligand or in combination with relaxin (acting on RXFP1) in rat renal myofibroblasts. Therefore, AT1R-AT2R-RXFP1 crosstalk can occur in matrix-producing myofibroblasts, where relaxin can mediate their anti-fibrotic effects in hypertensive kidneys. Harada H and colleagues suggested that P2Y_2_ receptor activation could increase mesangial cell proliferation in rat glomerular mesangial cells [76].

In hypertension, renal fibrosis may be induced by ET-1 as hyperuricemia occurs in 25 to 40% of adult patients with untreated hypertension. Teresa and colleagues demonstrated that ET-1 played a predominant role in the development of renal fibrosis [77]. In vascular endothelial cells derived from ET-1-knockout mice, renal fibrosis and myofibroblast formation were attenuated on days 7 and 14 after unilateral ureteral obstruction [78]. The activation of ET_A_ receptors drives the EMT in AngII-dependent hypertension and human renal tubular cells, which is associated with the Rho kinase signaling pathway and Yes-associated protein (Yap) [79].

## 3. GPCRs in Diabetic Nephropathy

**Figure 2:**
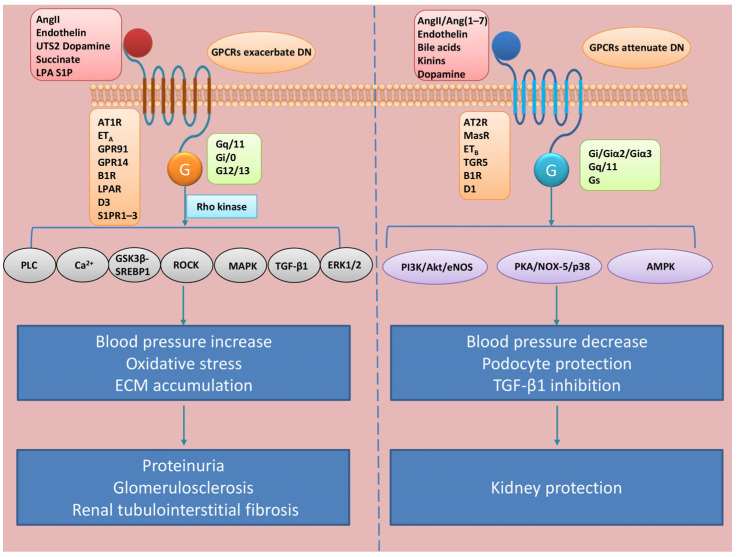
GPCRs in the pathogenesis of DN. The left panel shows pathogenic pathways. AngII, endothelin, UTS2, dopamine, succinate, and LPA S1P activate receptors including AT1R, ET_A_, GPR91, GPR14, B1R, LPAR, D3, and S1PR1–3, which couple with G-protein subtypes (Gq/11, Gi/0, G12/13) to drive downstream effector molecules such as Rho kinase, PLC/Ca^2+^, GSK3β-SREBP1, ROCK, MAPK, TGF-β1, and ERK1/2. These pathways increase blood pressure, oxidative stress, and ECM accumulation, ultimately leading to proteinuria, glomerulosclerosis, and renal tubulointerstitial fibrosis. The right panel shows protective pathways. These entail AngII/Ang(1–7), endothelin, bile acids, kinins, and dopamine-activating receptors, including AT2R, MasR, ET_B_, TGR5, B1R, and D1. These receptors couple with Gq/11, Gi/Giα2/Giα3, and Gs proteins to activate the PI3K/Akt/eNOS, PKA/NOX-5/p38, and AMPK pathways. Simultaneously, they suppress TGF-β1 signaling, exerting antihypertensive effects and podocyte-protective actions to mitigate renal damage.

GPCRs are involved in all stages of DN pathogenesis via regulating metabolism, blood pressure, immunity, and other risk factors (Figure 2). 

### 3.1. GPCRs in RAS

Building upon the roles of AT1R and AT2R described in hypertensive kidney damage, recent evidence suggests that the dysregulation of this receptor axis under hyperglycemic conditions contributes uniquely to the progression of diabetic nephropathy. RAS plays a critical role in the progression of DN, which has been well documented [80]. Although human clinical data for DN are conflicting, the experimental models show a consistent activation of RAS. Furthermore, RAS inhibition has been proven to be the single most effective therapeutic strategy for slowing the progression of DN in humans [81]. The activation of AT1R by AngII promotes ROS generation, activates MAPK, and facilitates TGF-β1 production, which accelerates glomerulosclerosis, podocyte loss, and tubulointerstitial fibrosis [10,82]. More than 20 years ago, AT1R antagonists were used to treat both patients with type 2 diabetes and rats with streptozotocin (STZ)-induced diabetes, and they were proven to be effective. In diabetes, the vasoconstriction of renal afferent arterioles is exaggerated in response to AngII. Like in HKD, AT2R and MasR have the opposite effects on the progression of DN compared to AT1R. The expression of AT2R is downregulated in both STZ-induced diabetic rats and spontaneously hypertensive rats with long-term diabetes. Many studies have demonstrated that the activation of AT2R or MasR has beneficial effects on DN [83,84]. Furthermore, some researchers suggest that the activation of AT2R or MasR produces better results than AT1R blockage [85]. A recent study indicates that using both an AT2R or MasR agonist, Ang(1–7), and an angiotensin-converting enzyme inhibitor has a positive add-on effect in diabetic nephropathy [55].

### 3.2. ET Receptors

Recently, research has focused on the ET antagonist added for RAS blockade to treat DN, with good results. Although several cardiovascular adverse events happened in patients with DN after 4 months of treatment, avosentan combined with RAS blockage treatment significantly reduced albuminuria in the early stages [86]. The excess cardiovascular adverse events were deemed to be due to the inhibition of ET_B_ because of the low selectivity for ET_A_ compared to ET_B_ (300:1). After that, another highly selective ET_A_ antagonist, atrasentan (selectivity for ET_A_ to ET_B_ (1800:1)), was chosen to combine with RAS blockage in DN, which reduced albuminuria, similarly to mild edema [87]. Research on atrasentan is still ongoing, and its benefit on DN is still being determined. In a controlled clinical trial involving 211 adults with DN, atrasentan-treated patients showed significantly reduced albuminuria, blood pressure, cholesterol, and triglyceride levels, with no significant change in GFR [88]. However, another stricter clinical trial performed in over 2500 adults with DN showed no significant change; thus, the study of atrasentan for DN was terminated [89]. Although the selective antagonists of ET_A_ are a worthy pursuit for DN therapy, in a DN model of mice with podocyte-specific double knockout of ET_A_ and ET_B_, less albuminuria, glomerulosclerosis, and podocyte loss were observed [90]. In a mouse model of DN, ET-1 induced the release of heparanase, leading to the disruption of the glycocalyx and the occurrence of proteinuria. However, by knocking out the ET receptors that were specifically expressed in podocytes, the expression of heparanase and the disruption of the glycocalyx can be significantly reduced, thereby improving proteinuria [91]. In animal models of diabetes, SLV306 (an inhibitor of endothelin-converting enzyme and neprilysin) could reduce proteinuria and prevent nephrosclerosis [92]. For treating hypertensive patients with type 2 diabetes, the inhibitors of RAS become less effective as the disease progresses; therefore, SLV306 provides an additional benefit of improving renal function by inhibiting the ET pathway.

### 3.3. GPR14

GPR14 is a receptor of urotensin II (UTS2), a potent mammalian vasoconstrictor peptide, and it has been reported to be abundantly expressed in both cardiovascular and renal tubular cells. Moreover, the expression of GPR14 is upregulated in DN in humans. The activation of GPR14 leads to a [Ca^2+^]i increase in the kidneys, which activates PLC and PLA2 [93]. In the heart, the activation of GPR14 is proven to promote cell proliferation and stimulate ECM accumulation [94,95]. A single-nucleotide polymorphism (SNP) study performed in a Hong Kong Chinese population with hypertension and diabetes indicated that UTS2 and GPR14 genes were associated with pancreatic β-cell function and insulin resistance but not hypertension [96]. In mice with early stage diabetes, UTS2 was demonstrated to induce ER stress and EMT, which increased ECM production in tubular epithelial cells. In addition, UTS2 and GPR14 could interact with AngII and TGF-β1 to promote their expression, which led to ECM synthesis and accumulation in DN [97]. UTS2 was found to be associated with oxidative stress in early DN. Although many UT receptor antagonists have been developed, their efficacy is not satisfactory. For example, two UT receptor antagonists, KR-36676 and KR-36996, have shown certain effects in inhibiting the proliferation of vascular smooth muscle cells in vitro and the formation of new tissues in vivo, but their effects on improving oxidative stress have not been clearly defined [98]. Though it shows positive effects on albuminuria and glycated hemoglobin in rat models, palosuran, a UTS2 receptor antagonist, is ineffective in hypertensive patients with DN [99]. Another more selective UTS2 antagonist, SB-6474510, was also found to have no effect on diabetic hyperglycemia [100]. The combination of urotensin II and GPR14 can exert biological effects such as vasoconstriction, cell proliferation, and the expression and secretion of the extracellular matrix. It regulates inflammatory signaling pathways like the JAK2/STAT3 pathway by inducing the production of profibrotic factors such as TGF-β1, and is involved in the occurrence and development of renal fibrosis. These studies indicate that UII is a key factor leading to renal fibrosis [101,102]. However, UTS2 receptor is still an attractive target of DN and the target of the top current standard-of-care DN treatments as it has neutral effects on hemodynamics, which contributes to lower side effects.

### 3.4. Takeda G-Protein-Coupled Receptor 5

Takeda G protein–coupled receptor 5 (TGR5; also known as GPBAR1, GPR131, M-BAR, and BG37) is a receptor of bile acids that regulates numerous metabolic pathways such as energy expenditure, glucose and lipid metabolism, and the modulation of immune responses [103,104]. TGR5 is expressed in many tissues, such as the intestine, gallbladder, adipose tissues, skeletal muscle, brain, and pancreas. TGR5 and another receptor of bile acids, farnesoid X receptor (FXR), complement each other for bile acid signaling mediation in kidney diseases. TGR5 can promote cAMP production as other GPCRs and affect mitochondrial genes, including sirtuin-3 (SIRT3), proliferator-activated receptor g coactivator 1-α (PPARα), and glucagon-like peptide-1 (GLP-1) [103]. With the progression of DN, the expression of TGR5 is downregulated at both the mRNA and protein levels in humans. In the DN model, the activation of TGR5 can significantly alleviate renal fibrosis and reduce the expression of fibrosis markers such as type I collagen, fibronectin, and TGF-β1 [105]. In addition, TGR5 agonists reduce the deposition of the extracellular matrix and inhibit the activation and proliferation of fibroblasts by suppressing the TGF-β1/Smad2/3 signaling pathway [106]. In high-glucose-treated rat glomerular mesangial cells (GMCs), TGR5 downregulated the expression of fibronectin and TGF-β1 by inhibiting RhoA/ROCK signaling via PKA [107]. In addition, TGR5 activation prevents high glucose-induced fibrosis in GMCs by suppressing the S1P/S1P2 signaling pathway.

### 3.5. GPR91

GPR91 originally was deorphanized as a receptor for succinate; thus, it was named SUCNR1. During hypoxia and metabolic stress, succinate is converted to fumarate by succinate dehydrogenase, also known as complex-II, which is capable of stabilizing HIF-1α and contributing to the physiological adaptation in hypoxia [108]. Importantly, the extracellular concentration of succinate increases along with different types of metabolic stress, such as hypertension, obesity, and diabetes. Therefore, GPR91 functions as a sensor of extracellular succinate to mediate autocrine or paracrine signals in metabolic stress. For example, in macrophages, the accumulation of succinic acid activates GPR91, which in turn promotes the production of IL-1β. This effect manifests as an autocrine signal. Meanwhile, GPR91 can also affect neighboring cells through a paracrine mechanism [109]. GPR91 is highly expressed in macula densa cells of the kidneys and is associated with many different diseases, including diabetic retinopathy and DN. Initially, some studies indicated that GPR91 was a major culprit in the pathogenesis of diabetes because the glucose tolerance and lipid metabolism of GPR91-deficient mice improved when they were fed a high-fat diet [110]. On the contrary, when GPR91 was specifically knocked out in the myeloid cells in mice, subcutaneous adipose tissue inflammation overall increased, leading to the exacerbation of the negative metabolic outcome [111]. It was also found that succinate accumulated locally in diabetic mouse kidneys and resulted in renin release using the juxtaglomerular apparatus [112]. In the renal tubules of diabetic mice, GPR91 was activated by succinate-induced ERK1/2 phosphorylation, which contributed to DN and tubulointerstitial fibrosis [113]. Thus, there is still a controversy about the beneficial versus damaging role of GPR91 in metabolic diseases.

### 3.6. GPCRs in the Kallikrein–Kinin System

The kallikrein–kinin system (KKS), a complex multienzyme system, plays an important role in DN, including circulating and tissue/renal KKS. There are two GPCRs, B1-receptor (B1R) and B2-receptor (B2R), that bind with kinins, such as bradykinin and kallidin [114]. Kinins in the kidneys mainly act in a paracrine or autocrine fashion. Although both receptors are linked to PLC activation and similar transduction pathways, they differ in terms of translational and transcriptional regulation. The B1R is induced in response to inflammatory stimuli such as endotoxins, lipopolysaccharides, and cytokines [115]. In diabetes, both receptors are activated due to oxidative stress, cytokines, and stimuli from other vasoactive peptides. In 1992, Harvey and colleagues reported that renal kallikrein excretion increased in patients with type 1 diabetes, which may be related to DN [116]. However, it has been demonstrated that human tissue kallikrein overexpression can prevent the development of DN in rats [117]. Treatment with the B2R antagonist, HOE-140, attenuated the beneficial effects of ACE inhibitors on albuminuria in type 1 and 2 diabetic models, suggesting that the activation of B2R is one of the underlying renoprotective mechanisms of ACE inhibitors [118]. Studies have verified the protective effects of KKS on rodent models with DN by promoting NO and prostaglandin production [119]. The downregulation of KKS components such as B2R and B1R induced apoptosis in the glomeruli of rats with STZ-induced diabetes and in high-glucose-treated podocytes. Thus, patients with diabetes and microalbuminuria presented reduced urinary kininogen-1 levels compared to those without microalbuminuria [120]. In addition, tissue kallikrein can efficiently prevent DN in STZ-induced diabetic rats by activating the insulin receptor, PI3K/Akt, AMPK, and MAPK signaling pathways [121]. In type 1 and 2 diabetic mice, pancreatic kallikrein treatment reduced proteinuria, renal inflammation, glomerulosclerosis, and oxidative stress via the activation of B1R and B2R [122]. Some studies also showed that ameliorating oxidative stress by KKS played a critical role in protecting DN through the synthesis of bradykinin and NO [123]. At all stages of DN, immune cells accumulate in the renal glomeruli and interstitial cells, resulting in the production of profibrotic, proinflammatory, and antiangiogenic factors. It has been reported that kallikrein reduced inflammatory cell accumulation in the tubulointerstitial and vasculature of salt-induced hypertensive rats [124]. In db/db mice, kallistatin overexpression suppressed renal inflammation in part by inhibiting TGF-β and NF-κB signaling pathways in the kidneys [125]. On the contrary, the activation of B1R enhances NF-κB-mediated cytokine synthesis, which promotes inflammatory response in DN [126]. The functions of B1R and B2R seem to be opposite, whereas the antagonists might exert beneficial effects in DN. The activation of KKS has shown great potential in preventing or reversing the progression of DN in animal models.

### 3.7. Lysophosphatidic Acid Receptors

Lysophosphatidic acid (LPA), a bioactive phospholipid, activates multiple cellular signaling pathways involved in various biological functions, such as cell proliferation, migration, and apoptosis, through binding to its receptors, LPAR1–6, which belong to CPCRs [127]. The six receptors are further grouped into two groups according to their distinct protein homology. LPAR1 to LPAR3 belong to the endothelial differentiation gene family, while LPAR4 to LPAR6 belong to the P2Y purinergic gene cluster. LPAR1 and LPAR2 can promote cell proliferation, survival, and migration by activating the Rho protein family of GTPases, PLC, MAPK, diacylglycerol (DAG), and PI3K/Akt via coupling with Gαi/o, Gαq/11, and Gα12/13. LPAR3 participates in LPA-induced Ca^2+^ mobilization, PLC, adenylyl cyclase inhibition, and MAPK activation by coupling with Gαi/o and Gαq/11 [128]. LPAR4 and LPAR5 trigger stress fiber formation and neurite retraction through the Rho/ROCK pathway via Gα12/13. LPAR4 can also increase the intracellular cAMP level by coupling with Gs. LPAR5 couples with Gαq/11 and increases the intracellular Ca^2+^ level [129]. LPAR6 is associated with cellular morphology through the Rho signaling pathway by coupling with Gαi/o or Gα12/13 [130]. The signaling pathways induced by the activation of these GPCRs are almost proven to be associated with the pathogenesis of DN. At the end of the 1990s, it was found that the circulation LPA levels were related to renal dysfunction in patients with renal failure [131]. Moreover, many studies demonstrated that LPA plays an important role in the progression of CKD induced by various pathogenic factors [132]. In eNOS (-/-) db/db mice, a robust model of DN, the LPA level was elevated in glomeruli. Recently, LPAR1 and/or LPAR3 were proven to be upregulated in different DN mouse models, and treatment with a dual-LPAR1/3 antagonist ameliorated renal functions by regulating the LPA–GSK3β–SREBP1 axis [133]. Mesangial cell proliferation and accumulation is a major risk factor in the pathogenesis of DN, which results in glomerulosclerosis. It was reported that LPA treatment induced the proliferation of mesangial cells by activating the Rac1/MAPK/KLF5 signaling pathway in DN [134]. Though the currently available data suggest that LPA promotes the progression of DN by binding to its receptors and regulating renal fibrosis and inflammation or inducing apoptosis, further studies should be performed to verify the mechanism of LPA.

### 3.8. Sphingosine-1-Phosphate Receptors

The sphingosine-1-phosphate (S1P) receptors (S1PRs) belonging to CPCRs, including S1PR1/EDG1, S1PR2/EDG5, S1PR3/EDG3, S1PR4/EDG6, and S1PR5/EDG8, mediate various physiological and pathophysiological processes by binding with extracellular S1P [135]. S1PR4 is mainly expressed in lymphoid tissues, whereas S1PR5 is restricted to the brain and spleen. S1PR1–3 are ubiquitous and contribute to the regulation of DN. The three S1PRs couple to different G proteins to initiate different intracellular signals: S1PR1 couples to Gi/o; S1PR2 most efficiently couples to G12/13 and also to Gi/o, Gs, and Gq; and S1P3 most efficiently couples to Gq and also couples to Gi/o and G12/13 [136,137]. The S1P/S1PR signaling pathway participates in renal fibrosis. It has been reported that the activation of S1PR2 induced the EMT in renal tubular epithelial cells through the activation of Rho kinase [138]. Inhibiting S1P/S1PR signaling showed renoprotective effects and downregulated fibrotic markers in a mouse model of DN, whereas S1P induced the expression of fibrotic markers, including α-SMA, in rat kidney fibroblast cells (NRK-49F cells). High glucose stimulation can activate the SphK1-S1P signaling pathway, thereby upregulating the expression of fibronectin [139]. In addition, S1P is essential for the TGF-β1 receptor-mediated activation of Smad signaling and stimulates the transdifferentiation of fibroblasts into myofibroblasts through S1PR3 activation [140]. The activation of S1PR2 and S1PR3 stimulates Rho/Rho kinase signaling, which plays a role in the differentiation of myofibroblasts. In high-glucose-treated renal glomerular endothelial cells, an S1PR2 antagonist can modify the morphology and function of mitochondria through the RhoA/ROCK1/Drp1 signaling pathway.

## 4. Conclusions

HKD is regulated by various GPCRs associated with renal vascular injury, podocyte loss, glomerulosclerosis, and tubulointerstitial fibrosis. We summarized a few of them, excluding GPCRs that are involved in hypertension and have the ability to participate in the regulation of HKD. The GPCRs that we mentioned are all regulators of blood pressure and act by binding with their ligands. However, blood pressure control can only slow down the progression of HKD, and most CKD patients ultimately develop ESRD. Deciphering the mechanisms that explain why CKD progresses even when blood pressure is under control is particularly critical. GPCRs are the most pursued drug targets because of their explicit structures and signal transmission modes. Furthermore, GPCRs interact with many other receptors and pathways, making them key factors in treating multiple diseases such as HKD. GPCRs involved in glucose and lipid metabolism, immunity regulation, insulin signaling mediation, hypertension, etc., might play a role in the pathogenesis of DN. We have selected some well-studied GPCRs to illustrate that GPCRs are major regulators of DN. Many researchers are committed to finding a combination therapy that can treat diseases with complex pathogeneses, using different medicines affecting multiple pathogenic factors. GPCRs remain some of the most desirable targets.

## Data Availability

No new data were created or analyzed in this study.

## Abbreviations

The following abbreviations are used in this manuscript:

HKD	hypertensive kidney disease
CKD	chronic kidney disease
GPCR	G-protein-coupled receptor
DN	diabetic nephropathy
ESRD	end-stage renal disease
VEC	vascular endothelial cell
VSMC	vascular smooth muscle cell
ECM	extracellular matrix
HIFα	hypoxia-inducible factor α
ATR	angiotensin II receptor
MasR	Mas receptor
MrgD	Mas-related G-protein-coupled receptor member D
RXFP	relaxin family receptor
AR	adenosine receptor
ETR	endothelin receptor
RAS	renin–angiotensin system
AngII	angiotensin II
PLC	phospholipase C
PLD	phospholipase D
PLA	phospholipase A
MAPK	MAP kinases
ROS	reactive oxygen species
NOS	nitric oxide synthase
NO	nitric oxide
MKP	MAPK phosphatase
PP2A	protein phosphatase 2A
PI3K	phosphoinositide 3-kinase
SHR	spontaneously hypertensive rat
GFR	glomerular filtration rate
MLC	myosin light chain
IP3	inositol 1,4,5-trisphosphate
ENaC	epithelial sodium channel
ET	endothelin
RSK	ribosomal S6 kinase
PKA	protein kinase A
Hhip	hedgehog interacting protein
[Ca^2+^]i	increased cytosolic Ca^2+^ concentration
EMT	epithelial–mesenchymal transition
ERK1/2	extracellular signal-regulated kinase 1/2
MMP	matrix metalloproteinase
Yap	Yes-associated protein
STZ	streptozotocin
SNP	single-nucleotide polymorphism
UTS2	urotensin II
TGR5	Takeda G protein-coupled receptor 5
FXR	farnesoid X receptor
SIRT	sirtuin
PPARα	proliferator-activated receptor g coactivator 1-α
GLP-1	glucagon-like peptide-1
GMC	glomerular mesangial cell
KKS	kallikrein–kinin system
LPA	lysophosphatidic acid
DAG	diacylglycerol
S1P	sphingosine-1-phosphate
S1PR	S1P receptor
